# Cytoplasmic, full length and novel cleaved variant, TBLR1 reduces apoptosis in prostate cancer under androgen deprivation

**DOI:** 10.18632/oncotarget.9005

**Published:** 2016-04-26

**Authors:** Garrett Daniels, Xinmin Zhang, Xuelin Zhong, Larion Santiago, Ling Hang Wang, Xinyu Wu, Jack Y. Zhang, Fengxia Liang, Xin Li, Thomas A. Neubert, Laurey Steinke, Ying Shen, Ross Basch, Robert Schneider, David E. Levy, Peng Lee

**Affiliations:** ^1^ Department of Pathology, New York University School of Medicine, New York, NY, USA; ^2^ Department of Pathology and Laboratory Medicine, Hofstra North Shore-LIJ School of Medicine, Hempstead, NY, USA; ^3^ Department of Basic Science and Craniofacial Biology, New York University College of Dentistry, New York, NY, USA; ^4^ Department of Biochemistry and Molecular Pharmacology, New York University School of Medicine, New York, NY, USA; ^5^ Department of Biochemistry and Molecular Biology, University of Nebraska Medical Center, Omaha, NE, USA; ^6^ Department of Microbiology and Molecular Pathogenesis, New York University School of Medicine, New York, NY, USA; ^7^ Department of Urology, New York University School of Medicine, New York, NY, USA; ^8^ NYU Cancer Institute, New York University School of Medicine, New York, NY, USA; ^9^ New York Harbor Healthcare System, New York University School of Medicine, New York, NY, USA

**Keywords:** prostate cancer, subcellular localization, castration resistance, TBLR1, cvTBLR1

## Abstract

TBLR1/TBL1XR1, a core component of the nuclear receptor corepressor (NCoR) complex critical for the regulation of multiple nuclear receptors, is a transcriptional coactivator of androgen receptor (AR) and functions as a tumor suppressor when expressed in the nucleus in prostate. Subcellular localization of a protein is critical for its function, and although TBLR1, as a transcriptional cofactor, has been primarily viewed as a nuclear protein, many cells also express variable levels of cytoplasmic TBLR1 and its cytoplasmic specific functions have not been studied. Prostate cancer (PCa) cells express moderately higher level of cytoplasmic TBLR1 compared to benign prostate cells. When comparing androgen-dependent (AD) to androgen-independent (AI) PCa, AI cells contain very high levels of TBLR1 cytoplasmic expression and low levels of nuclear expression. Overexpression of cytoplasmic TBLR1 in AD cells inhibits apoptosis induced by androgen deprivation therapy, either in an androgen free condition or in the presence of bicalutamide. Additionally, we identified a cytoplasmic specific isoform of TBLR1 (cvTBLR1) approximately 5 kDa lower in molecular weight, that is expressed at higher levels in AI PCa cells. By immunoprecipitation, we purified cvTBLR1 and using mass spectrometry analysis combined with N-terminal TMPP labeling and Edman degradation, we identified the cleavage site of cvTBLR1 at amino acid 89, truncating the first 88 amino acids of the N-terminus of the full length protein. Functionally, cvTBLR1 expressed in the cytoplasm reduced apoptosis in PCa cells and promoted growth, migration, and invasion. Finally, we identified a nuclear export signal sequence for TBLR1 cellular localization by deletion and site-directed mutagenesis. The roles of TBLR1 and cvTBLR1 provide novel insights into the mechanism of castration resistance and new strategies for PCa therapy.

## INTRODUCTION

Prostate cancer (PCa) is an exceedingly prevalent cancer and is the second leading cause of cancer related death of men in the US [1]. It has long been known that androgens are critical for PCa growth and androgen ablation therapy is still the mainstay of treatments for patients with metastatic PCa. Although initially effective, these cancers inevitably recur as castration resistant prostate cancer (CRPC) for which there is still no reliable treatment. Deaths from PCa are primarily due to the development of castration resistant metastatic disease. Many mechanisms of CRPC have been proposed and studied, including but not limited to increased Androgen Receptor (AR) expression, crosstalk with other signaling pathways, AR splice variants, and variable AR cofactor expression [2–5].

Transducin beta like related 1 (TBLR1), also known as TBL1XR1, is a core component of the nuclear receptor corepressor (N-CoR) and is a silencing mediator of retinoic acid and thyroid receptor (SMRT) complex [6, 7]. A secondary isoform of TBLR1 (TBLR1β) has been previously identified, formed from alternative splicing creating a variable C-terminus and a slightly larger protein of about 60 kDa [8]. As an intrinsic component of these corepressor complexes, TBLR1 has essential functions both as a corepressor and a coactivator of multiple nuclear receptors [9, 10]. Additionally, TBLR1 and the closely related TBL1X are necessary for activation of Wnt target genes [11]. TBL1X plays an important role in protecting beta catenin from degradation in the cytoplasm allowing for transcription of its targets genes [12]. Overexpression of TBLR1 inhibits growth in 293T and Jurkat cells and is involved in degradation of the anti-apoptotic protein BCL-3 [8, 13]. TBLR1 is a ubiquitously expressed protein [14]. Immunohistochemical data shows TBLR1 as primarily a nuclear protein in normal prostate but shows reduced nuclear expression in PCa [15]. TBLR1 is also a strong coactivator of AR in prostate cancer, leading to selective activation of growth suppressive AR target genes and tumor suppression. In contrast to the reduced expression of TBLR1 in PCa, expression of TBLR1 is upregulated in breast, colon, esophageal squamous cell and nasopharyngeal carcinomas (NPC) and is a potential prognostic marker for aggressive cervical cancer [16–18]. Overexpression of TBLR1in NPC cells causes reduced sensitivity to cisplatin-induced apoptosis [18].

Nuclear transport is critical for controlling localization and proper function of proteins. Many proteins exhibit different functions based on their post-translational modifications and cellular compartmentalization. The AR coactivator p44/MEP50 displays a tumor suppressor role in the nucleus but promotes PCa growth and prostate development when expressed in the cytoplasm [19]. Transport of proteins across cellular compartments is regulated at several protein and cellular levels [20], including nuclear localization signals (NLS) and nuclear export signals (NES). Common NLS sequences contain arginine and lysine, and NES sequences are leucine rich, however these cases do not appear to be universal [21, 22].

In this study we characterize the expression and function of cytoplasmic TBLR1, which is found in a 55 kDa full length form and, in PCa, in a 50 kDa isoform also. We found increased cytoplasmic TBLR1 in CRPC and also identified a post-translational cleaved variant of TBLR1 localized only in the cytoplasm that is also higher in CRPC. We found that TBLR1 when localized in the cytoplasm inhibits apoptosis induced by androgen ablation in PCa cells. Additionally, we identified a nuclear export sequence within the protein necessary for its sub-cellular compartmentalization. This study may provide insight to develop effective targets for the treatment of CRPC.

## RESULTS

### Reduction of apoptosis by full length cytoplasmic TBLR1 (cTBLR1) under androgen deprivation

**Figure 1:**
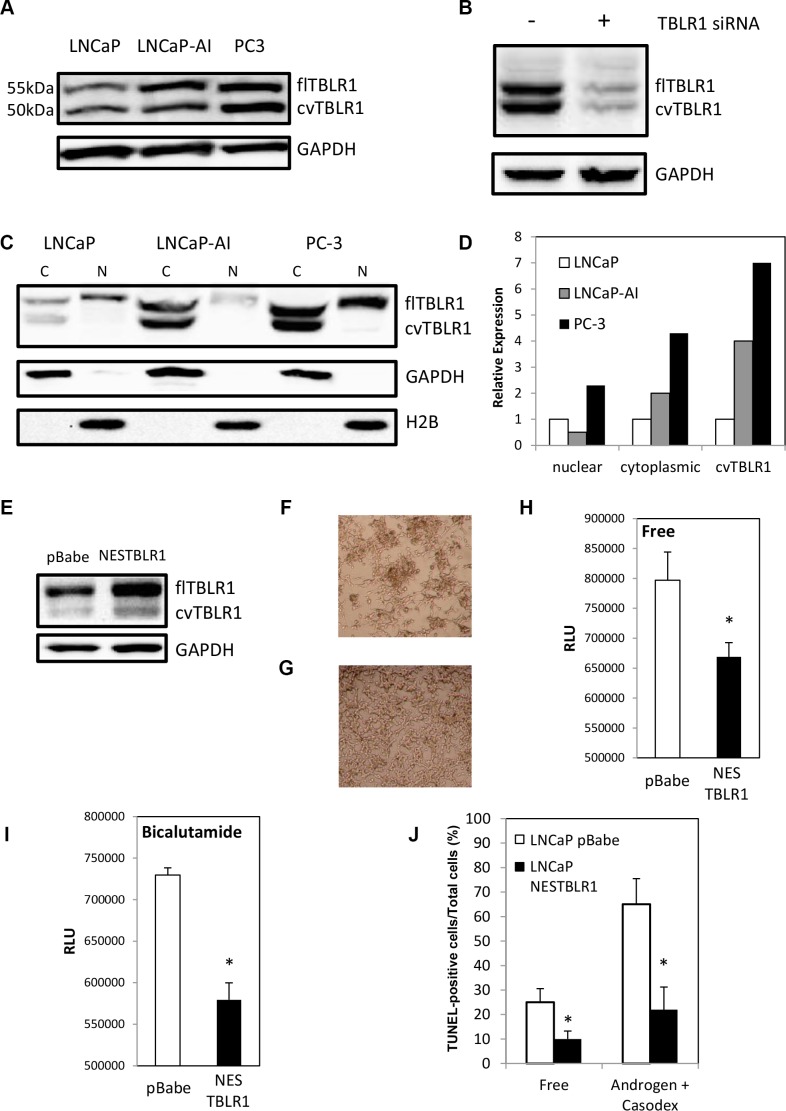

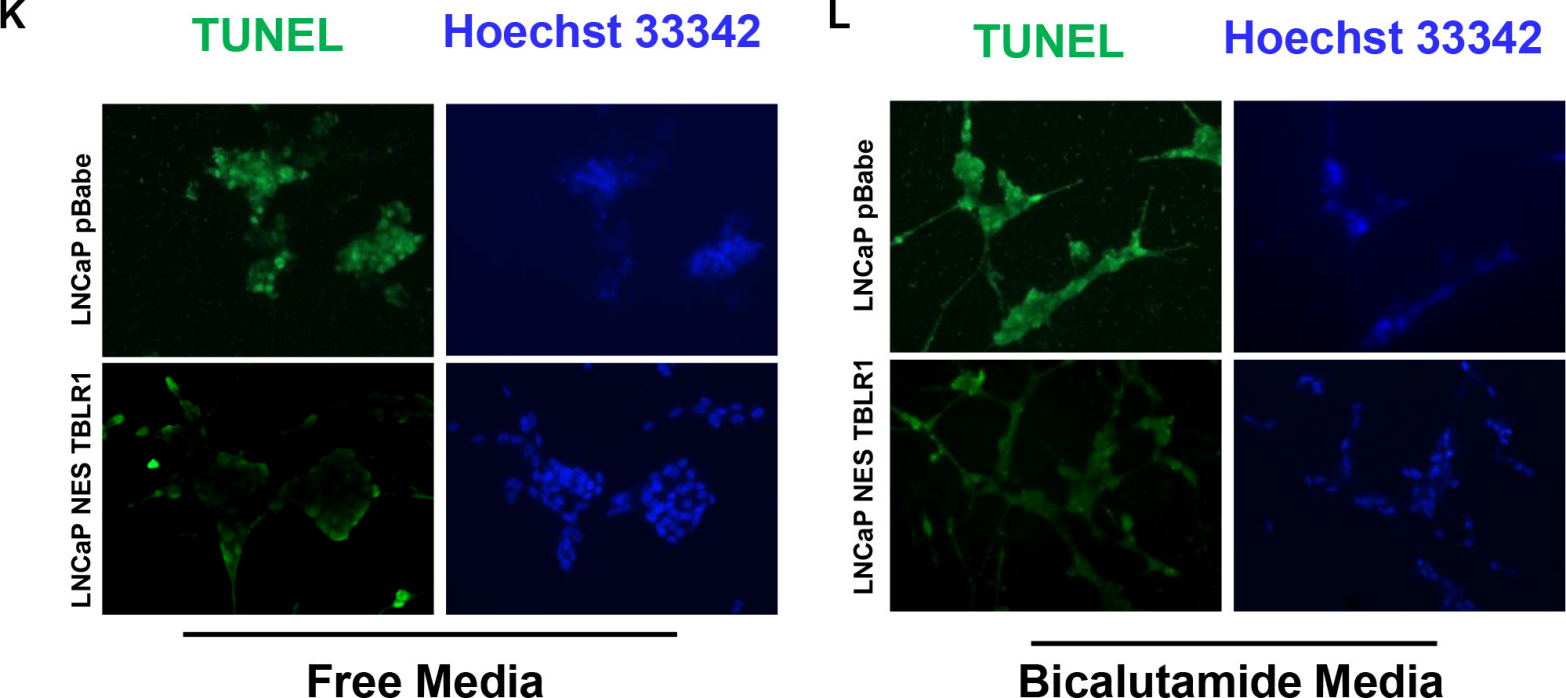
Expression of full length cytoplasmic TBLR1 (cTBLR1) and protease cleaved cytoplasmic variant of TBLR1 (cvTBLR1) in prostate cancer cells (**A**) Relative total expression of TBLR1 and cvTBLR1 in androgen-dependent LNCaP cells compared to androgen-independent LNCaP-AI and PC-3 cells. (**B**) Confirmation of faster migrating band (cvTBLR1) as a TBLR1 variant by knockdown with TBLR1 specific siRNA in LNCaP-AI cells. (**C**) Relative expression and localization of TBLR1 and cvTBLR1 in prostate cancer cells by western blot of fractionated lysates. (**D**) Quantification of fractionation western blot comparing localization and relative expression of TBLR1 and cvTBLR1 in prostate cancer cells. (**E**) Overexpression of cytoplasmic TBLR1 using nuclear export sequence (NES) fused TBLR1 construct to create LNCaP –NESTBLR1 cells. (**F**) LNCaP pBabe cells after 6 days in hormone free media. (**G**) LNCaP NESTBLR1 cells after 6 days in hormone free media. (**H**) Caspase 3/7 luciferase assay measuring apoptosis in LNCaP pBabe and LNCaP NESTBLR1 cells in hormone free media. (**I**) Caspase 3/7 luciferase assay measuring apoptosis in LNCaP pBabe and LNCaP NESTBLR1 cells in 150 μM bicalutamide media. (**J**) Quantification of the percentage of TUNEL-positive cells of LNCaP pBabe vs. LNCaP NESTBLR1 cells in hormone free media or 150 μM bicalutamide media. (**K**) TUNEL staining on LNCaP control vs. LNCaP NESTBLR1 cells in androgen free and androgen media with (**L**) 150 μM bicalutamide treatment.

Previously our lab showed that TBLR1 was expressed as both nuclear and cytoplasmic protein [15]. In PCa cells, nuclear expression of TBLR1 was significantly reduced in comparison to benign prostate cells. In this study we compared cytoplasmic expression of TBLR1 and its function between androgen-dependent (AD) PCa cells and androgen-independent (AI) PCa cells. When comparing TBLR1 levels by western blot between AD LNCaP PCa cell lines and two AI PCa cell lines, LNCaP-AI and PC-3, we found increased total level of TBLR1 in the AI cells (Figure 1A). To determine the subcellular localization of the increased TBLR1 in AI cancer cell lines compared to AD cancer cells, we performed western blot analyses with fractionated nuclear and cytoplasmic cell lysates. We found that the increased level of TBLR1 in AI cancer cell lines was due to increased cytoplasmic TBLR1 (Figure 1C, 1D).

Previously our lab showed that overexpression of nuclear TBLR1 in AD LNCaP cells inhibited growth in an androgen-dependent manner by cell cycle arrest but this overexpression had no effect on apoptosis [15]. To test the function of cytoplasmic TBLR1, we created stable LNCaP cell lines overexpressing TBLR1 in the cytoplasm utilizing a fusion construct of the nuclear export sequence (NES) (MLQKKLEELE) to the N-terminus of TBLR1. We confirmed overexpression and cytoplasmic localization of TBLR1 in these cells by western blot of total lysate (Figure 1E) and fractionated lysate (Supplementary Figure 1A). LNCaP cells require androgens for survival and in their absence, these cells undergo apoptosis [23]. LNCaP NESTBLR1 cells exhibited reduced cell death visually (Figure 1G) in androgen free media compared to control cells (Figure 1F). Next, we performed Caspase 3/7 activity assays to test the effect of cytoplasmic TBLR1 on apoptosis in androgen-free and regular media with and without bicalutamide, an anti-androgen medication. LNCaP NESTBLR1 cells showed significant reduction of Caspase 3/7 activity after 48 hours in androgen-free medium (Figure 1H). We also observed a significant reduction of Caspase 3/7 activity after 30 hours in 150 μm anti-androgen bicalutamide media in LNCaP NESTBLR1 cell (Figure 1I). We confirmed reduced apoptosis in androgen-free conditions and bicalutamide-treated cells in LNCaP NESTBLR1 cells compared to LNCaP pBabe cells by TUNEL assay. Quantification of TUNEL-positive cells showed approximately 2.5 fold reduction for LNCaP NESTBLR1 cells in both conditions (Figure 1J). Representative pictures showing reduced TUNEL staining in both conditions in LNCaP NESTBLR1 cells are shown in Figure 1K–1L. Notably, we also tested cell proliferation and migration/invasion ability between LNCaP pBabe and LNCaP NESTBLR1 cells and we did not observe any difference (data not shown). The reduction in apoptosis by NESTBLR1 in regular medium by both assays was not statistically significant (data not shown). In cell proliferation, migration and invasion assays, NESTBLR1 did not show statistically significant differences when compared to control.

### Identification and function of 50 kDa cytoplasmic-specific TBLR1 isoform

In addition to full length TBLR1, we identified a faster migrating protein band recognized by TBLR1 antibody that runs at approximately 50 kDa (Figure 1A). We consistently observed cvTBLR1 across multiple cell lines including LNCaP-AI, C4-2B and VCaP. To confirm this second observed band as a variant of TBLR1 protein, we transfected LNCaP-AI cells with specific siRNA for TBLR1. TBLR1 specific siRNA reduced expression of both full length and the faster migrating variant. Conversely, overexpression of full length TBLR1 cDNA by transfection into cells also led to increased cvTBLR1 by western blot (data not shown). These data confirm the 50 kDa band is a specific TBLR1 isoform (cvTBLR1) (Figure 1B). We also found that cvTBLR1 was only expressed in the cytoplasm and was significantly increased in AI cells (Figure 1C). Over expression of NES-TBLR1 also leads to increased cvTBLR1 levels in the cytoplasm (Supplementary Figure 1A).

**Figure 2:**
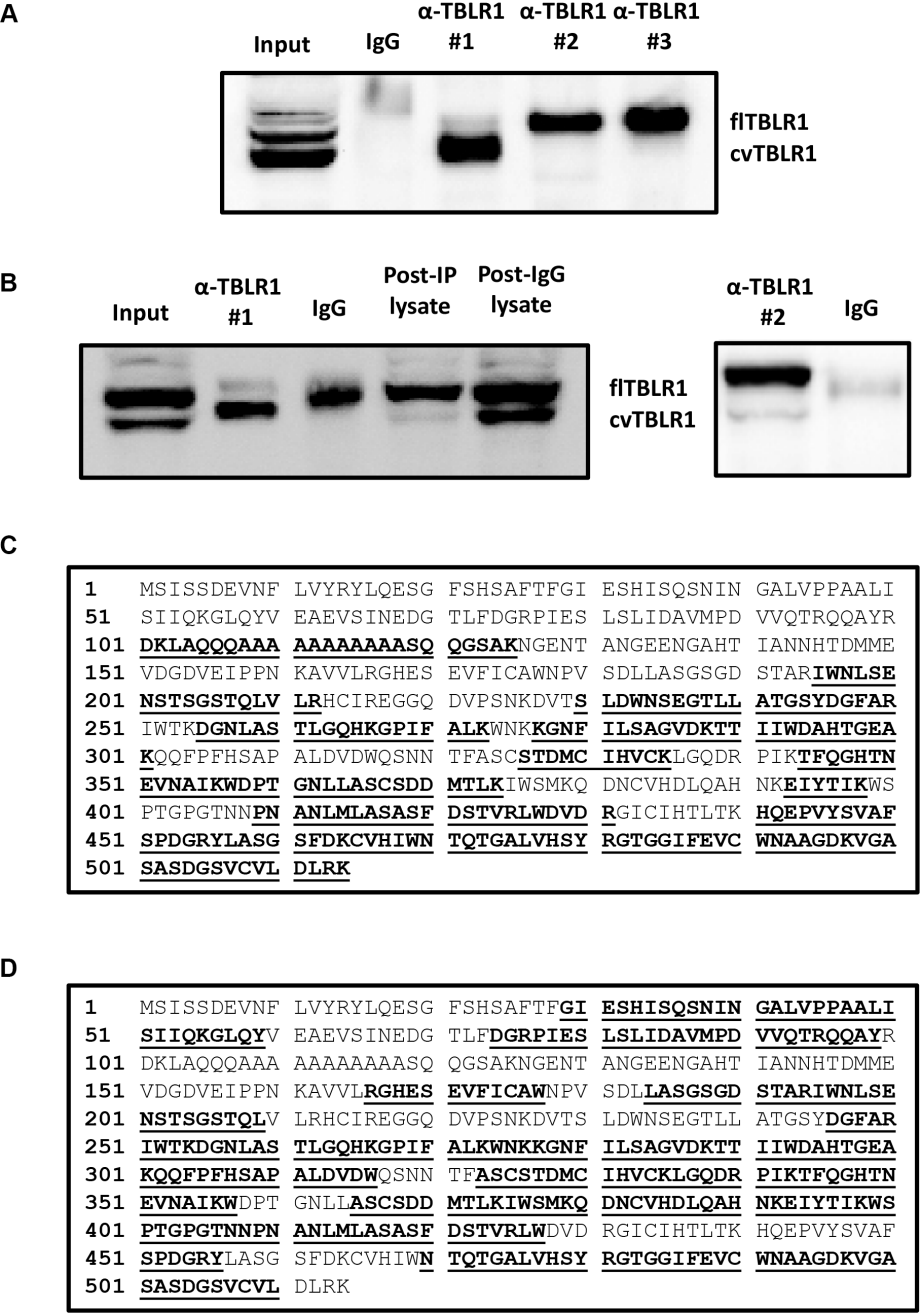

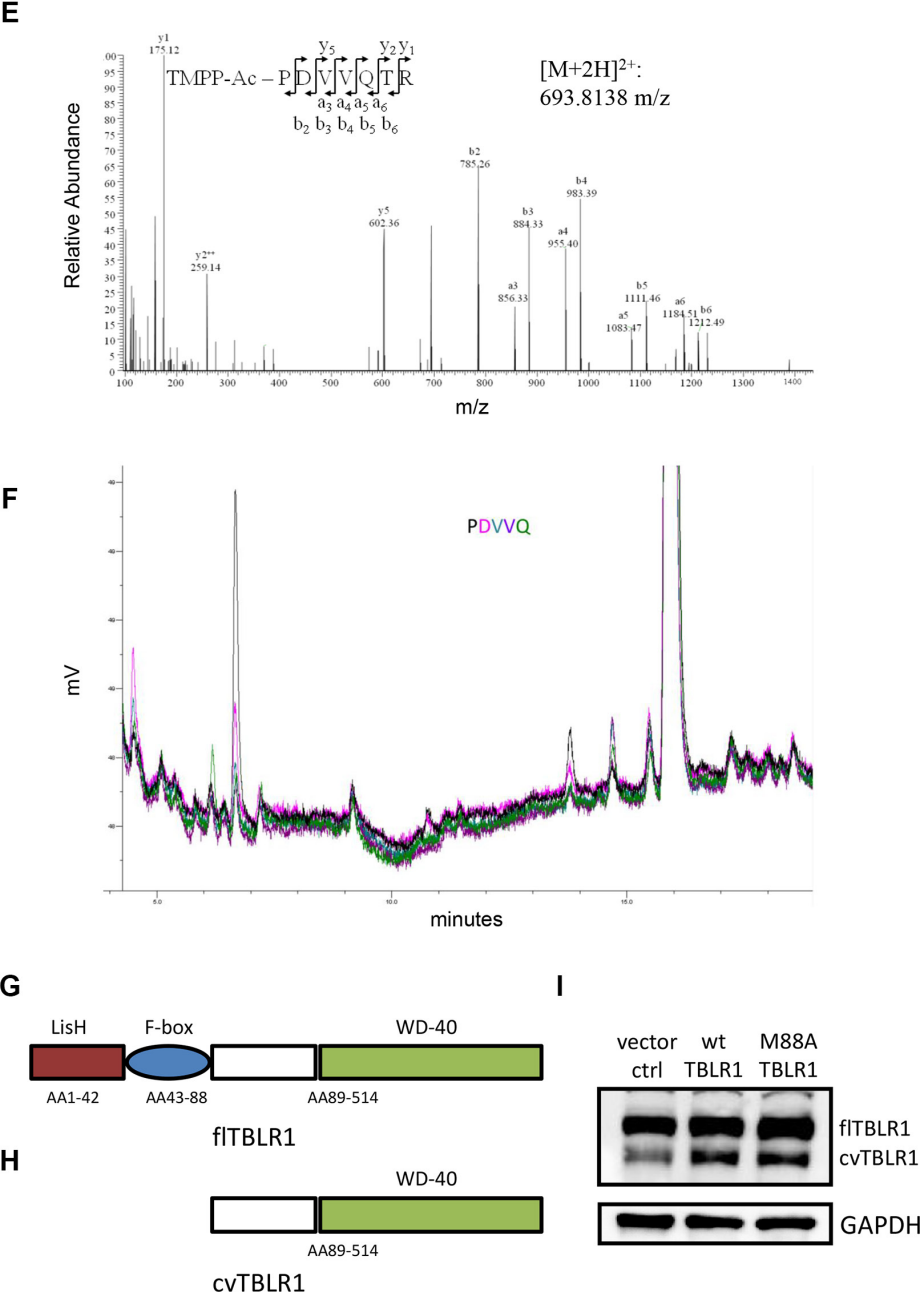
Sequence identification of cvTBLR1 (**A**) Immunoprecipitation with three different TBLR1 specific antibodies to identify antibody to purify cvTBLR1. (**B**) Large scale purification of cvTBLR1 using antibody #1 and full length TBLR1 using antibody #2 for mass spectrometry (MS) sequencing. (**C**) TBLR1 sequence showing bold/highlighted sequences of peptides identified by MS from purified cvTBLR1. (**D**) TBLR1 sequence showing bold/highlighted sequences of peptides identified by MS from purified full length TBLR1. (**E**) Identification of N-terminal sequence of cvTBLR1 using TMPP labeled purified cvTBLR1 followed by MS sequence analysis. MS/MS spectrum of the doubly charged N-terminal peptide PDVVQTR of cvTBLR1 is shown, with the peptide sequence with annotation of the matched ions above the spectrum. (**F**) Confirmation of N-terminus of cvTBLR1 by Edman degradation analysis of the protein, overlay plot of the first five cycles. Y axis is millivolts. Absorbance is measured at 269 nm. Color code for the chromatogram matches the colors of the text reporting the identified amino acids. (**G**) Diagram of conserved domains in full length TBLR1. (**H**) Diagram of cvTBLR1 lacking the LisH and F-box domains of full length TBLR1 important for transcriptional regulation. (**I**) Overexpression of wildtype or mutated M88A full length TBLR1 shows increased cvTBLR1 in both conditions.

In order to isolate and purify cvTBLR1 for analysis we tested different commercial TBLR1 antibodies for TBLR1 immunoprecipitation to identify potential capture antibodies specific for cvTBLR1. We used three different TBLR1 specific antibodies and found that each antibody selectively pulls down a single form of TBLR1 (Figure 2A). Interestingly, all three of these antibodies recognize both forms by western blot (data not shown). Antibody #1, a monoclonal antibody designed from peptide sequence AA 81-179 of the 514 AA protein specifically immunoprecipitated cvTBLR1 with only small amounts of full length TBLR1 present. We next prepared large-scale immunoprecipitation preps in PC-3 cells using either Antibody #1 or Antibody #2 for purification. Pulldown of cvTBLR1 was first confirmed by western blot (Figure 2B). Antibody #1 primarily pulled down cvTBLR1 and post-TBLR1 IP lysate showed reduced levels of cvTBLR1 compared to post-IgG IP, confirming a very efficient capture. Colloidal coomassie blue stain was performed on purified protein to be processed by mass spectrometry (MS) analysis (Supplementary Figure 1B).

Purified cvTBLR1 and full length TBLR1 were analyzed by tryptic digestion and MS to identify and compare known peptide sequences present. cvTBLR1 sequence lacked evidence of the first 100 amino acids suggesting this region of full length TBLR1 could be lacking in this isoform (Figure 2C). No peptides from the first 100 amino acids were detected by MS in three separate cvTBLR1 purifications (data not shown). We next performed MS analysis of purified full length TBLR1 to determine if detection of these peptides was not readily detected by our mass spectrometer. This analysis of full length TBLR1 revealed the presence of additional peptides up to AA 28, supporting the conclusion that this N-terminal region was lacking in cvTBLR1 (Figure 2D). To identify the exact location of the cleavage to form cvTBLR1, we next utilized in-gel TMPP N-terminus labeling of purified cvTBLR1 prior to tryptic digestion followed by MS analysis. MS analysis can recognize the presence of the TMPP label and identify the N-terminus sequence of the original protein. We found PDVVQ to be the N-terminus labeled peptide with P corresponding to amino acid 89 of TBLR1 (Figure 2E). We confirmed these findings by N-terminal Edman degradation of purified cvTBLR1 that also found P89 as the N-terminus of cvTBLR1 (Figure 2F). This N-terminus region contains the LisH domain critical for histone binding for transcriptional corepression and the F-box like domain for transcriptional coactivation (Figure 2G, 2H). Since AA88 of TBLR1 is a methionine, there was a strong possibility that cvTBLR1 could be the product of alternate translation. To test for alternate translation, we created a mutant construct of TBLR1 altering the methionine at amino acid 88 into an alanine (M88A) and stably transfected the mutant and control plasmid into 293T cells. In both conditions we identified increased cvTBLR1 which rules out alternate translation as a method of cvTBLR1 production (Figure 2I). Additionally, there is no evidence of an alternate mRNA spliced variant of this size. Together this data suggests that cvTBLR1 could be a proteolytic cleavage of full length TBLR1 or a potential alternate translation product.

**Figure 3:**
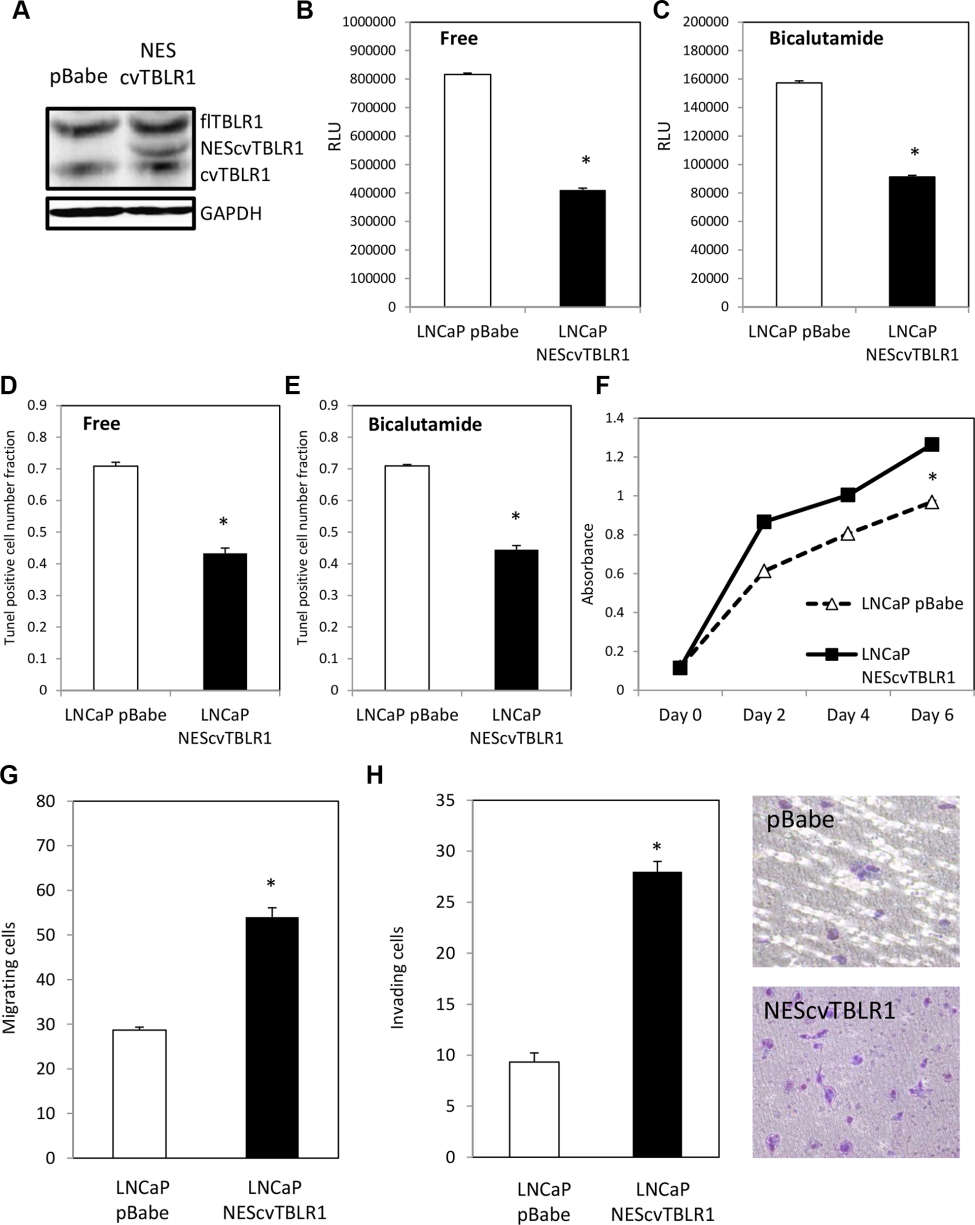
Function of cvTBLR1 (**A**) Overexpression of cvTBLR1 using nuclear export sequence (NES) fused cvTBLR1 (AA89-514) construct to create LNCaP –NEScvTBLR1 cells. (**B**) Caspase 3/7 luciferase assay measuring apoptosis in LNCaP pBabe and LNCaP NEScvTBLR1 cells in hormone free media. (**C**) Caspase 3/7 luciferase assay measuring apoptosis in LNCaP pBabe and LNCaP NEScvTBLR1 cells in 150 μM bicalutamide media. (**D**) Quantification of the percentage of TUNEL-positive cells of LNCaP pBabe vs. LNCaP NEScvTBLR1 cells in hormone free media. (**E**) Quantification of the percentage of TUNEL-positive cells of LNCaP pBabe vs. LNCaP NEScvTBLR1 cells in 150 μM bicalutamide media. (**F**) Cellular proliferation comparison of LNCaP pBabe vs. LNCaP NEScvTBLR1 cells over 6 days by CCK-8 assay. (**G**) Migration assay of LNCaP-NEScvTBLR1 compared to LNCaP pBabe control. (**H**) Invasion assay of LNCaP-NEScvTBLR1 compared to LNCaP pBabe control and representative pictures of membrane.

We next tested the function of cvTBLR1 in prostate cancer cells. We created a fusion construct in the pBabe retroviral vector of nuclear export sequence (NES)-cvTBLR1 (AA89-514 of TBLR1) and stably transfected it into LNCaP prostate cancer cells (Figure 3A). We tested the effect of cvTBLR1 on apoptosis in these cells by a Caspase 3/7 assay and found significant reduction of Caspase 3/7 activity in androgen-free and bicalutamide media signifying a reduction in apoptosis (Figure 3B, 3C). We confirmed these findings by TUNEL assay in androgen-free media and bicalutamide media (Figure 3D, 3E). Interestingly and in contrast to full length TBLR1, overexpression of cvTBLR1 also led to a significant increase in cellular proliferation over a 6-day growth assay (Figure 3F). We compared migration and invasion ability by measurement of cells traversing either an uncoated porous membrane (migration) or matrigel-covered membrane (invasion) using serum as a chemoattractant. We found significantly more migrating and invading cells with overexpression of cvTBLR1 (Figure 3G, 3H).

### Mutation analysis to identify nuclear export sequences (NES) in TBLR1

**Figure 4:**
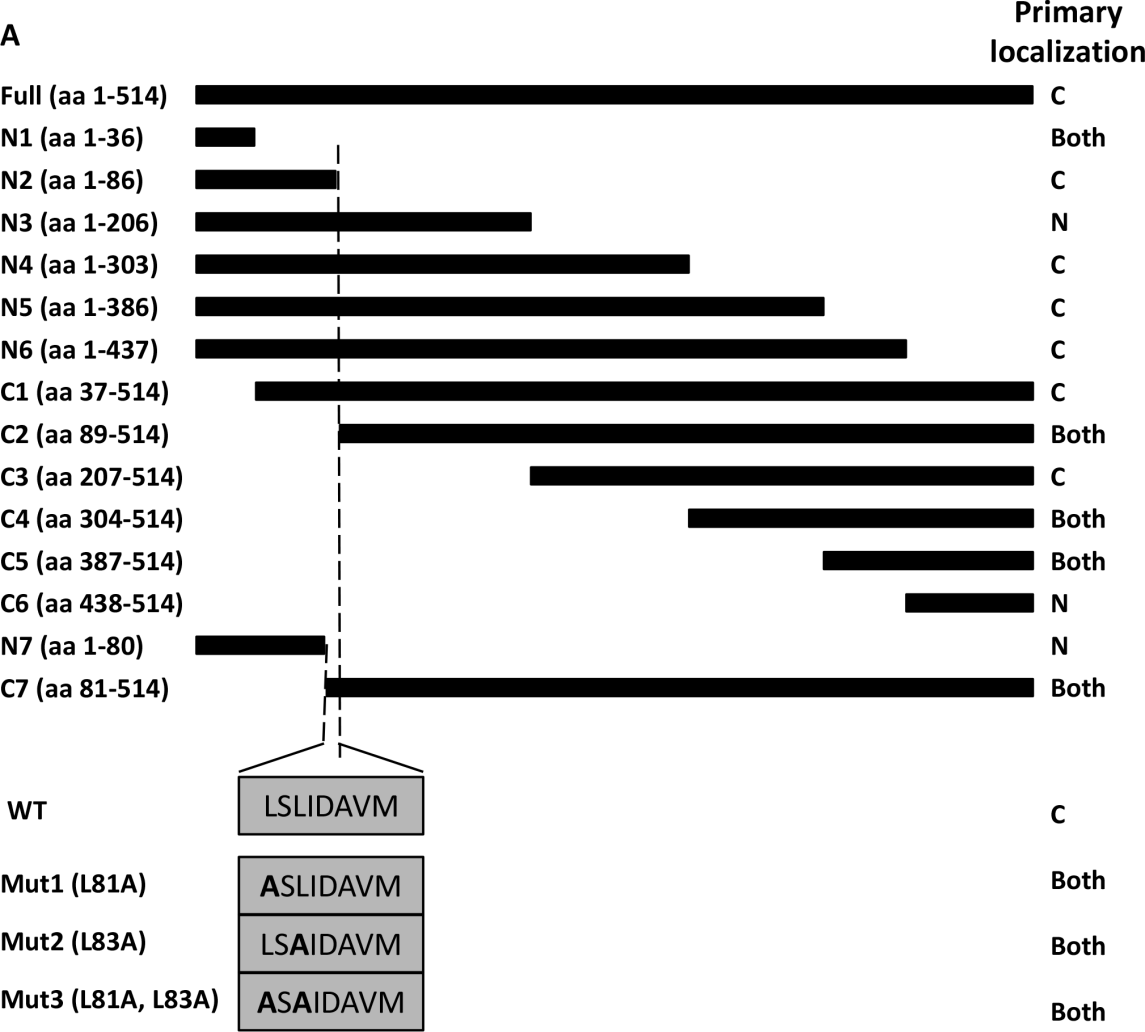

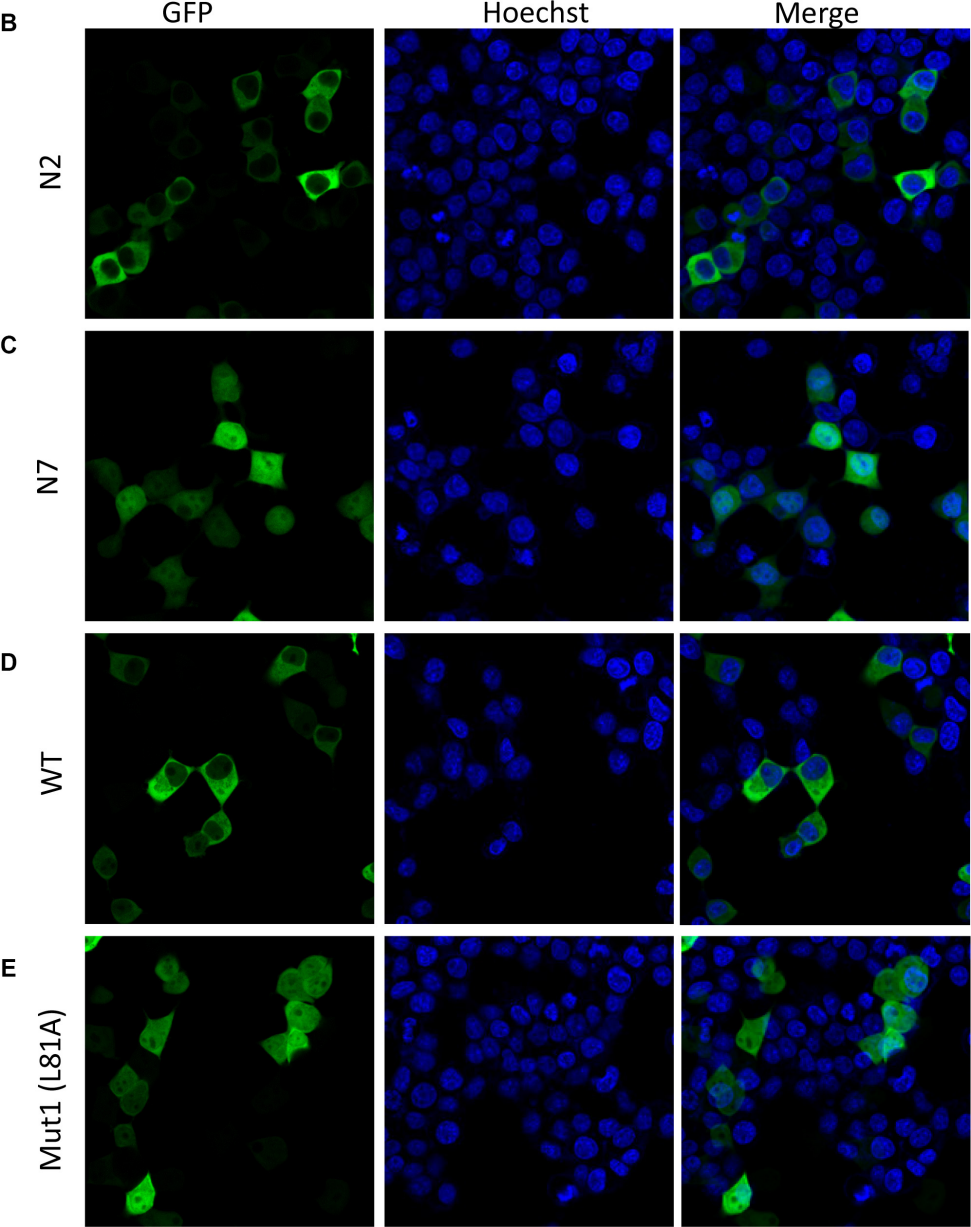

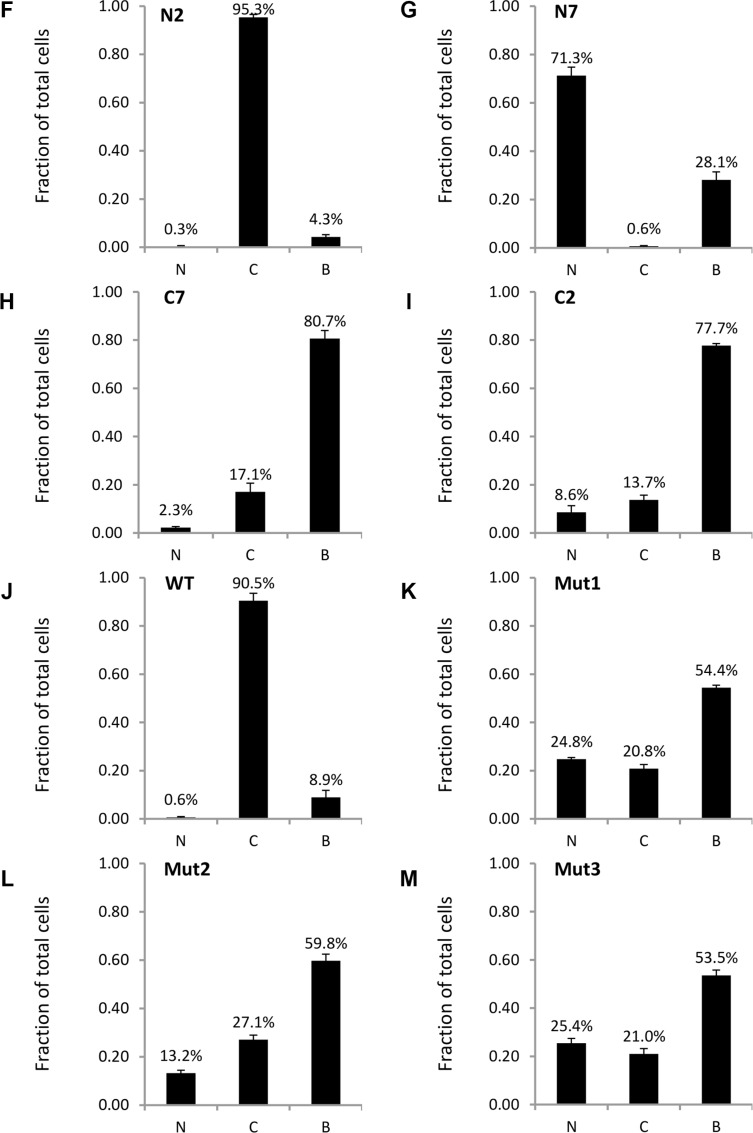
Identification of TBLR1 localization sequences by deletion mutation analysis (**A**) Diagram of GFP-TBLR1 mutant constructs designed and summary of primary localization of transfected GFP fusion mutant construct. (**B–E**) Fluorescence image, Hoescht stain, and merged image of 293T cells transfected with GFP-TBLR1 mutant constructs N2, N7, wild type, and mutant L81A. (**F–M**) Quantification of TBLR1 localization observed in 293T cells transfected with mutant GFP-TBLR1 constructs. Cells showing GFP expression by fluorescent imaging were counted as cytoplasmic, nuclear, or both for expression.

To identify potential nuclear export sequences (NES), we created a series of N-terminal (C1-C7) and C-terminal (N1-N7) deletion mutants. Additionally, we used site-directed mutagenesis to alter Leucine 81 (mut1) or Leucine 83 (mut2) or both (mut3) to alanine. We cloned these constructs into a pCDNA-GFP vector to visualize localization by confocal microscopy after transient transfection into 293T cells (Figure 4A).

After transfection, cells were counted as nuclear, cytoplasmic, or both (equal expression). Figure 4A summary shows the predominant localization for each construct. Cells transfected with N2 construct expressing amino acid (AA) 1–86 of the 514 AA wild type (WT) TBLR1 showed strong cytoplasmic expression (Figure 4B, 4F). Cells transfected with N7 (AA1-80), however, showed stronger nuclear expression (Figure 4C, 4G). Interestingly, although endogenous TBLR1 is expressed in both the cytoplasm and nucleus of cells, overexpression of GFP-TBLR1 shows very strong cytoplasmic expression (Figure 4D, 4J). Since NES sequences are often leucine-rich [22] and there are two leucines within the AA81-86 region, we used site-directed mutagenesis to mutate each of these leucines in the full length TBLR1 (mut1, mut2, mut3) and transfected these constructs into 293T cells. Transfection of mut1 no longer showed strong cytoplasmic localization of WT, but rather more equal distribution (Figure 4E, 4K). Mut2 and mut3 also showed nuclear predominant localization (Figure 4L–4M). Quantification of the number of cells with nuclear or cytoplasmic TBLR1 localization of additional constructs is located in Supplementary Figure 2. Together this data confirms AA81 and AA83 are important residues for a strong NES in TBLR1.

## DISCUSSION

TBLR1 was originally identified as an integral component of the NCOR/SMRT co-repressor complex. Subsequently, it was found that TBLR1 can also act as a transcriptional activator for nuclear hormone receptors and regulated transcription factors. TBLR1 is overexpressed in several types of cancer and functions as an oncogene by activation of important intracellular signaling pathways including the Wnt-β-catenin pathway and the nuclear factor-kappa (NF-kB) pathway [11, 24]. TBLR1 expression has a positive correlation with disease progression, tumor metastasis, and drug resistance [16–18, 25–27]. Our previous study has shown that TBLR1 is a transcriptional activator of AR and its nuclear expression is significantly reduced in PCa compared with benign prostate glands. Forced expression of nuclear TBLR1 leads to androgen-dependent growth suppression of prostate cancer cells *in vitro* and *in vivo* by selective activation of androgen-regulated genes associated with differentiation and growth suppression [15]. In addition to its nuclear localization, TBLR1 can also be a cytoplasmic protein. TBLR1 is predominantly located in the cytoplasm of LNCaP-AI cells in complete media but serum starvation induces its translocation into nucleus. These findings suggest that cytoplasmic TBLR1 may play an important role distinct from that of nuclear TBLR1. However, the functionality in most past TBLR1 studies did not clarify if the effects were due to nuclear or cytoplasmic TBLR1.

In this study, we found that the total level of TBLR1 protein is increased in AI PCa cell lines compared to an AD PCa cell line. For example, cytoplasmic TBLR1 is expressed at a higher level in both LNCaP-AI and PC-3 cells compared to LNCaP cells. We also identified a novel TBLR1 variant-cvTBLR1. It is exclusively located in cytoplasm and expressed at a much higher level in AI PCa cells than in AD PCa cells. In theory, the cvTBLR1 could be the product of alternative splicing, alternative translation or proteolytic cleavage. However, it is very interesting that when we stably overexpressed full length TBLR1 cDNA, PCa cells also showed increased presence of cvTBLR1. This provided evidence against formation of cvTBLR1 by alternate splicing and evidence for post-translational formation. Due to cvTBLR1 pro-proliferative and anti-apoptotic roles, stable overexpression of TBLR1 may lead to selective pressure on cells to increase formation of cvTBLR1. This appears to be a slow process, since we have not observed a noticeable increase in cvTBLR1 with transient overexpression of full length TBLR1.

We were able to isolate cvTBLR1 by taking advantage of an interesting observation that the TBLR1 antibodies we used to purify cvTBL1 exhibited different specificities. While all TBLR1 antibodies detected both forms on western blot, the TBLR1 antibody from Abcam preferentially pulled down only cvTBLR1 by immunoprecipitation. A possible explanation is that cvTBLR1 has a tertiary structure different from that of full length TBLR1, which makes certain epitopes accessible for this antibody. As a result, this change may be responsible for the localization and functions of cvTBLR1. Mass spectrometric analysis of the TMPP-labeled purified cvTBLR1protein showed that it lacks the first 88 amino acids of TBLR1. To support that cvTBLR1 is a product of proteolytic cleavage, we identified by Edman degradation the N-terminus of cvTBLR1 as PDVVQ, a peptide sequence located between amino acid 89 and 93 of TBLR1. This site is conserved in proteolytic cleavage sites [28]. There is a methionine at amino acid 88 of TBLR1 suggesting that cvTBLR1 could be a product of alternative translation. We created a TBLR1 mutant that has an alanine replacing the methionine at amino acid 88, destroying the second translation initiation site. When the mutant is expressed, the levels of full-length TBLR1 and cvTBLR1 are still both increased, which provides strong evidence that cvTBLR1 is not due to alternative translation.

Our previous study showed that overexpression of nuclear TBLR1 inhibited LNCaP cell growth in an androgen-dependent manner by cell cycle arrest but had no effect on apoptosis [15]. In contrast, this study demonstrates that overexpression of cytoplasmic TBLR1, both full length and cvTBLR1, significantly reduces apoptosis when measured by both fluorescent Caspase 3/7 assays and Tunel experiments, consistent with recent reports suggesting TBLR1 mediates antiapoptotic effects [18]. Most significantly, the inhibition of apoptosis is more dramatic in the androgen-deprived condition, either in androgen-free medium or in the presence of the anti-androgen, bicalutamide. These findings suggest distinct functions of nuclear and cytoplasmic TBLR1.

TBLR1 contains a conserved F-box-like domain that associates with Skp1 in ubiquitin-dependent protein degradation. In *Drosophila* Ebi, the homolog of TBL1 and TBLR1, binds Tramtrack and promotes its degradation [29, 30]. The canonical Wnt/β-catenin pathway plays an important role in prostate tumorigenesis and is significantly mutated in CRPC. The level of β-catenin is regulated by two mechanisms: the adenomatous polyposis coli/Axin/glycogen synthase kinase 3 beta-dependent degradation pathway and the Siah-1/Siah interacting protein/Ebi-mediated degradation pathway. Ebi(ΔF), which lacks the F-box domain and retains ability to bind β-catenin, is capable of preventing the Sial-induced downregulation of β-catenin and TCF/LEF1 activity [31]. cvTBLR1 also lacks the N-terminal F-box like region of full length TBLR1. Given the high structural similarity between cvTBLR1 and Ebi(ΔF), the overexpression of cvTBLR1 in AI PCa cells suggest that cvTBLR1 may function as a dominant negative form to inhibit β-catenin degradation and thus induce cytoplasmic accumulation of β-catenin and TCF/LEF activity. Further, it was shown that TBL1/TBLR1 can bind β-catenin and the interaction is required for its translocation into the nucleus [11]. It is of great interest to investigate the link between cvTBLR1 and β-catenin and TCF/LEF1 pathway in future studies.

Finally, we identified a NES for cytoplasmic localization of TBLR1 at the N-terminus which contains two conserved leucine residues. By targeting these sites, we may be able to develop approaches to switch the cellular localization, thus the functions of TBLR1.

In conclusion, TBLR1 appears to be a multifunctional protein important for many cellular processes. In this study, we demonstrate that cytoplasmic TBLR1 is increased in CRPC cells. We also identify a novel TBLR1 variant that is potentially a proteolytic product, higher in CRPC, and promotes growth, invasion, and antiapotosis. Both full length and and cvTBLR1 may play a pivotal role in castration resistance of PCa. It will be of great interest to investigate the TBLR1 cytoplasmic forms in CRPC in the future.

## MATERIALS AND METHODS

### Cell culture

LNCaP, LNCaP-AI, PC-3, and 293T cells were maintained in complete medium (RPMI medium 1640 (High glucose DMEM for 293T) plus 10% FBS), androgen-free medium (phenol-free RPMI medium with charcoal-treated FBS), or androgen media (androgen-free medium with 10 nM R1881). LNCaP-AI, an androgen-independent derivative of LNCaP, was from the laboratory of Dr. A. Ferrari (New York University Cancer Institute) [32]. LNCaP, PC-3, and 293T cells were procured from ATCC (Manassas, VA, USA) with documentation of authentication by STR analysis. Passages from 5 to 20 of each cell line were used for experiments. Cell lines were maintained at 37°C with 5% CO_2_. All cell culture materials were ordered from Life Technologies.

### Whole cell lysates, cell fractionation, and immunoblot analysis

Whole-cell extracts were prepared from LNCaP, LNCaP-AI, and PC-3 cells in RIPA buffer (50 mM Tris-HCl ph 8.0, 150 mM NaCl, 1% NP-40, 1 mM EDTA, 0.1% SDS). For cell fractionation, cells were lysed with cytoplasmic buffer (10 mM HEPES ph 7.6, 50 mM NaCl, 0.5 M sucrose, 1 mM DTT, 5 mM MgCl_2_, 0.1% Triton X-100). After centrifugation, the supernatant was the cytoplasmic fraction and the nuclear pellet was washed 3 times with cytoplasmic buffer followed by lysis with RIPA buffer. Extracts were subjected to electrophoresis on SDS/PAGE and then transferred to nitrocellulose membranes for immunoblot analysis as described [33]. Antibodies for TBLR1#1 (sc-100908) were purchased from Santa Cruz antibodies. Antibodies for H2B and GAPDH were purchased from Cell Signaling. Antibodies for TBLR1#2 (ab24550) and TBLR1#3 (ab84141) were purchased from Abcam.

### Protein purification and edman degradation

Initial immunoprecipitation of TBLR1 was performed with three separate antibodies described above. Cells were lysed with 50 mM Tris pH 8.0, 150 mM NaCl, 1 mM EDTA, 0.5% NP-40 buffer for 20 minutes on ice. Lysates were clarified by centrifugation for 20 minutes at 13000 rpm at 4°C. Clarified lysates were incubated with 2 μg of TBLR1 or IgG control antibody overnight at 4°C, followed by addition of 20 μl Dynabeads Protein G (Invitrogen) washed with lysis buffer for 4 hours at 4°C. Immunoprecipitated material was washed 4× with lysis buffer and eluted with 2× Laemmli buffer at 37°C for 5 minutes. Large scale 50 kDa TBLR1 isoform purification by immunoprecipitation for mass spectrometry with and without TMPP labeling or Edman degradation for N-terminus analysis were performed as above except after clarification and before addition of antibody NaCl was added to bring final NaCl concentration to 300 mM to further purify immunoprecipitate. Protein gels for mass spectrometry were stained with Novex colloidal blue staining kit (Invitrogen) per manufacturer's intructions. For Edman degradation, protein gels were transferred to PVDF membrane as described previously [33], as membranes were stained by colloidal blue staining kit (Invitrogen). Protein transferred to PVDF was analyzed by automated Edman degradation in the Protein Structure Core Facility, University of Nebraska Medical Center, using a Procise 494 protein sequencer (ABI). All reagents were purchased from Life Technologies.

### TMPP labeling and mass spectrometry of TBLR1

Excised gel bands were reduced, alkylated and dried. Protein was then digested with trypsin (Promega) overnight at 37°C. Digested peptides were extracted, desalted and analyzed by LC-MS/MS (Q Exactive, Thermo Electron). Raw data were used to search with Mascot Distiller a small custom database containing 176 protein sequences including TBLR1. Protein N-terminal identification was performed as described in [34]. Ten microliters of 0.1 M of (N-succinimidyloxycarbonyl-methyl) tris (2, 4, 6-trimethoxyphenyl) phosphonium bromide (TMPP-Ac-OSu) (Sigma) in acetonitrile were added to gel pieces and incubated overnight. Protein was then digested with trypsin in TEAB buffer (Promega) overnight at 37°C. Digested peptides were extracted, desalted and analyzed by LC-MS/MS (Q Exactive, Thermo Electron). Raw data were searched without enzyme restriction.

### Construction of retroviral pBabe vectors expressing NESTBLR1 or NEScvTBLR1 to construct stable cell lines

A TBLR1 fusion protein was created in which the strong NES (MLQKKLEELE) was fused to the N terminus of TBLR1 to create NESTBLR1. An additional fusion protein was created in which the strong NES was fused to the sequence of AA89-514 from full length TBLR1 to create NEScvTBLR1. Both fusion proteins were cloned into the pBabe Puro retroviral vector as described previously [19]. Briefly, Phoenix A amphotropic packaging cells (American Type Culture Collection) were transfected with NESTBLR1, NEScvTBLR1 and pBabe retroviral constructs to produce virus. The virus-containing supernatant was collected by centrifugation and filtered before retroviral infection. Stable cell lines were selected in 1 μg/mL puromycin.

### Apoptosis assays

Promega luciferase Apoptosis was measured by Caspase-GLO 3/7 assay (Promega) on cells after treatment with 150 μm bicalutamide or DMSO control for 30 hours or hormone free or 10 nM R1881 for 72 hours. Luciferase readings were read on Berthold Lumat LB 9507. The TUNEL assay was performed using Click- iT^®^ TUNEL Alexa Fluor^®^Imaging Kit (Invitrogen) in accordance with the manufacturer's protocol. In brief, cells were fixed with 4% paraformaldehyde in PBS at room temperature for 20 min and permeabilized with Triton X-100 (0.25% in PBS) for another 20 min. The cells were washed twice and incubated with terminal deoxynucleotidyl transferase reaction buffer (Component A) for 10 min at room temperature. The buffer was removed and the TUNEL reaction mixture containing terminal deoxynucleotidyl transferase was added, incubating the cells in a humidified chamber at 37°C for 60 min. Then, cells were washed three times with 3% BSA in PBS for 2 min each and then incubated with Click-iT reaction mixture (containing Alexa 488 azide) for 30 min at room temperature, while being protected from light exposure. The cells were again washed with 3% BSA in PBS and the cell nuclei were counterstained with Hoechst 33342 for 15 min at room temperature, protected from light. The coverslips were washed twice with PBS before mounting onto a slide with mounting medium. The TUNEL-positive cells were counted in eight different, random fields for each well.

### Deletion and site-directed mutagenesis

TBLR1 C-terminus deletions (N1, N2, N3, N4, N5, N6 and N7), N-terminus deletions (C1, C2, C3, C4, C5, C6, C7 and C8), and both terminus deletions (M1 and M2), were generated by PCR and cloned into pCR2.1-TOPO vectors (TOPO TA Cloning kit, 450641, Life Technologies). These deletions were then subcloned into pcDNA3.1 (+) with GFP plasmid. pcDNA3.1 TBLR1 point mutations (Mut1, Mut2 and Mut3) were generated using QuickChange II XL Site-Directed Mutagenesis Kit (200521, Stratagene). Mut1 is Leu81Ala, Mut2 is Leu83Ala and Mut3 is Leu81&83Ala. QuickChange II XL was also used to create TBLR1 M88A mutant construct. The list of primers used to generate these clones in Supplementary Table 1.

### Confocal microscopy

To evaluate the intracellular localization of TBLR1 deletions and site-directed mutants, 293 cells were seeded in each well of 4 well chamber slides w/cover glass slide (154534, Thermo Fisher Scientific) and transfected with 0.5 μg DNA of GFP-TBLR1 constructs as indicated. At 24 hr, NucBlue Live Cell Stain ReadyProbes Reagent Hoechst33342 (R37605, Life Technologies) were added to the medium and cells were incubated for 20 min at 20– 25°C. Fluorescent images of live cells were obtained with a Zeiss LSM710 confocal microscope imaging system and analyzed with ImageJ software.

### Enumeration of percentage of cells for nuclear and cytoplasmic localization

293T cells were seeded in three wells of a 4 well chamber slide (155382, Thermo Fisher Scientific) and transfected with 0.5 ug DNA of GFP-TBLR1 constructs in triplicate. At 24 hr, subcellular localization of TBLR1 and mutants was examined with a Nikon Eclipse TS100 fluorescent microscope and a Nikon DXM1200F digital camera. A minimum of 100 cells per well were recorded from each of three wells. Localization in each individual cell was determined based on comparison of intensities between cytoplasmic and nuclear GFP, and therefore categorized into nuclear expression, cytoplasmic expression and both nuclear and cytoplasmic expression. Percentage of cells in each category was calculated. Results are expressed as mean + standard error.

### Growth, migration, and invasion assays

For growth assay, cells were seeded in 24 well plates at 1 × 10^4^ cells/well. Cell growth was monitored every 2 days by addition of 50 μl of CCK-8 reagent (Sigma Life Sciences) to the well, incubated at 37°C for 1 hour and absorbance was measured at 450 nm. For migration and invasion assays, cells (5 × 10^4^) were plated into Corning Biocoat Matrigel invasion chambers or chambers with no matrigel for migration in triplicate without serum. 10% FBS media was used as an attractant in the lower chamber and cells were incubated for 24 hours. Membranes were fixed, washed and stained with a Three Step Stain Kit (Richard-Allan Scientific) and three representative regions of each membrane were counted under the microscope.

### Statistical analysis

Statistical analyses of the above results were performed by pairwise Student's *t* test. Differences are considered statistically significant if *p* < 0.05.

## SUPPLEMENTARY MATERIALS FIGURES AND TABLE


