# Supplementary material for: Cytoplasmic, full length and novel cleaved variant, TBLR1 reduces apoptosis in prostate cancer under androgen deprivation
Source: Oncotarget. 2016 Apr 26;7(26):39556–71. doi: 10.18632/oncotarget.9005 (PMC5129953; doi:10.18632/oncotarget.9005)
Supplement: Supplementary file 1 [file oncotarget-07-39556-s001.pdf]

Cytoplasmic, full length and novel cleaved variant, TBLR1 reduces apoptosis in prostate cancer under androgen deprivation

Supplementary Materials

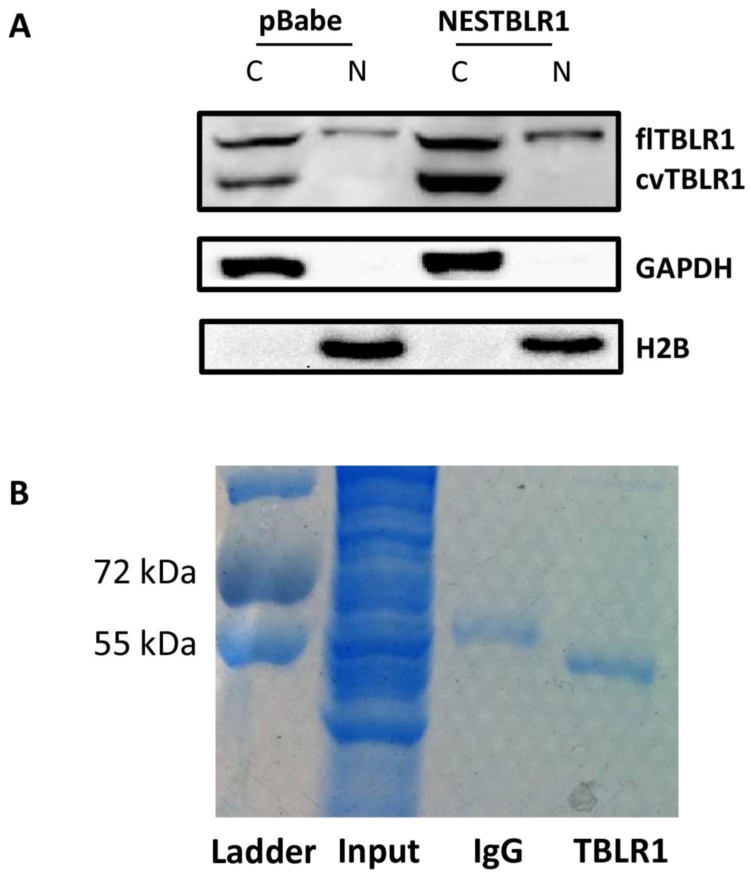

Supplementary Figure S1: Colloidal coomassie blue stain of cvTBLR1 purification. (**A**) Western blot for TBLR1 on fractionated cell lystates comparing LNCaP NESTBLR1 to LNCaP pBabe control. (**B**) Total cell lysate input and 10% loading of cvTBLR1 and IgG control immunoprecipitation for large scale purification of protein used for mass spectrometric anaylsis.

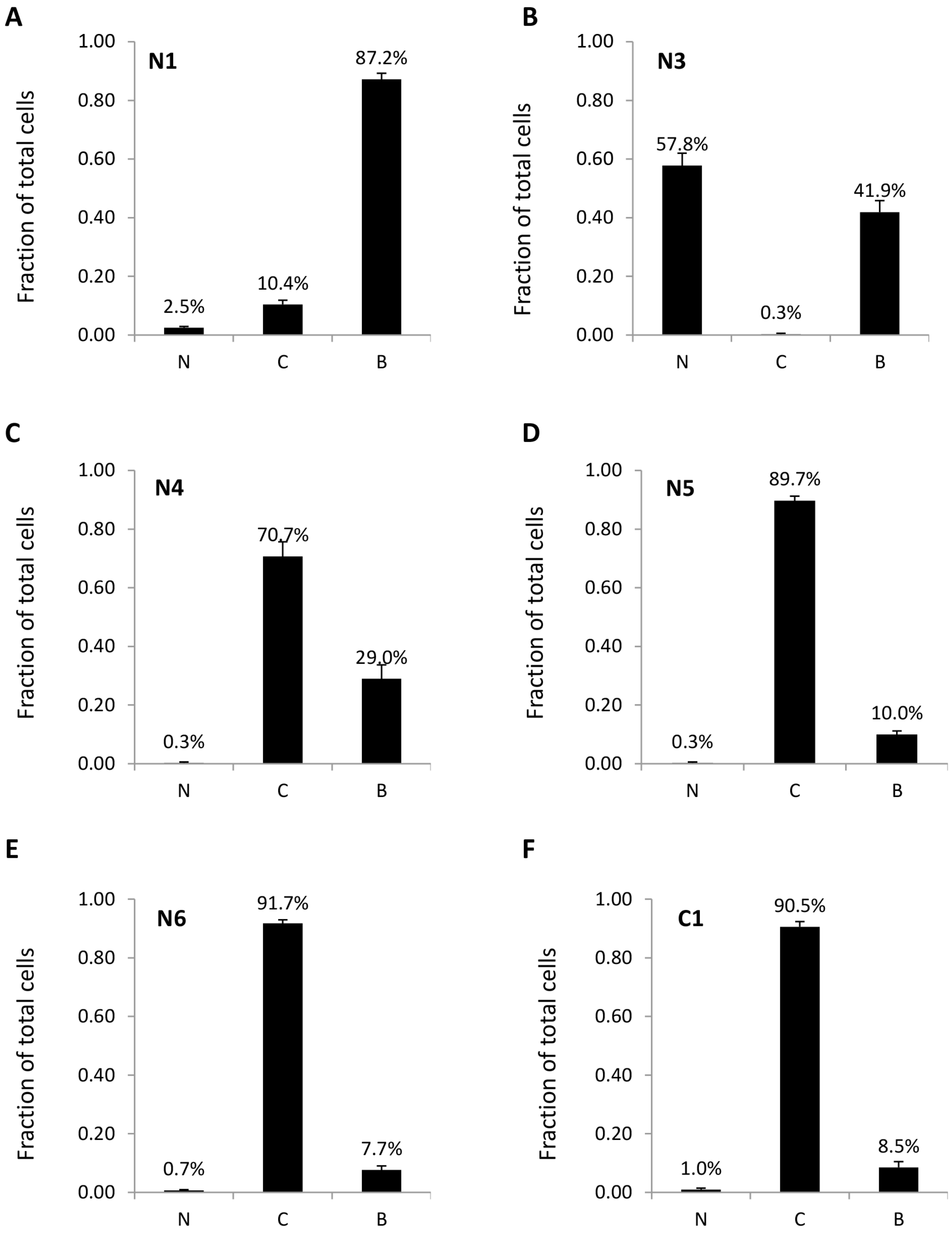

A
N1
Fraction of total cells
1.00
0.80
0.60
0.40
0.20
0.00
N
C
B
2.5%
10.4%
87.2%
B
N3
57.8%
0.3%
41.9%
C
N4
0.3%
70.7%
29.0%
D
N5
0.3%
89.7%
10.0%
E
N6
0.7%
91.7%
7.7%
F
C1
1.0%
90.5%
8.5%

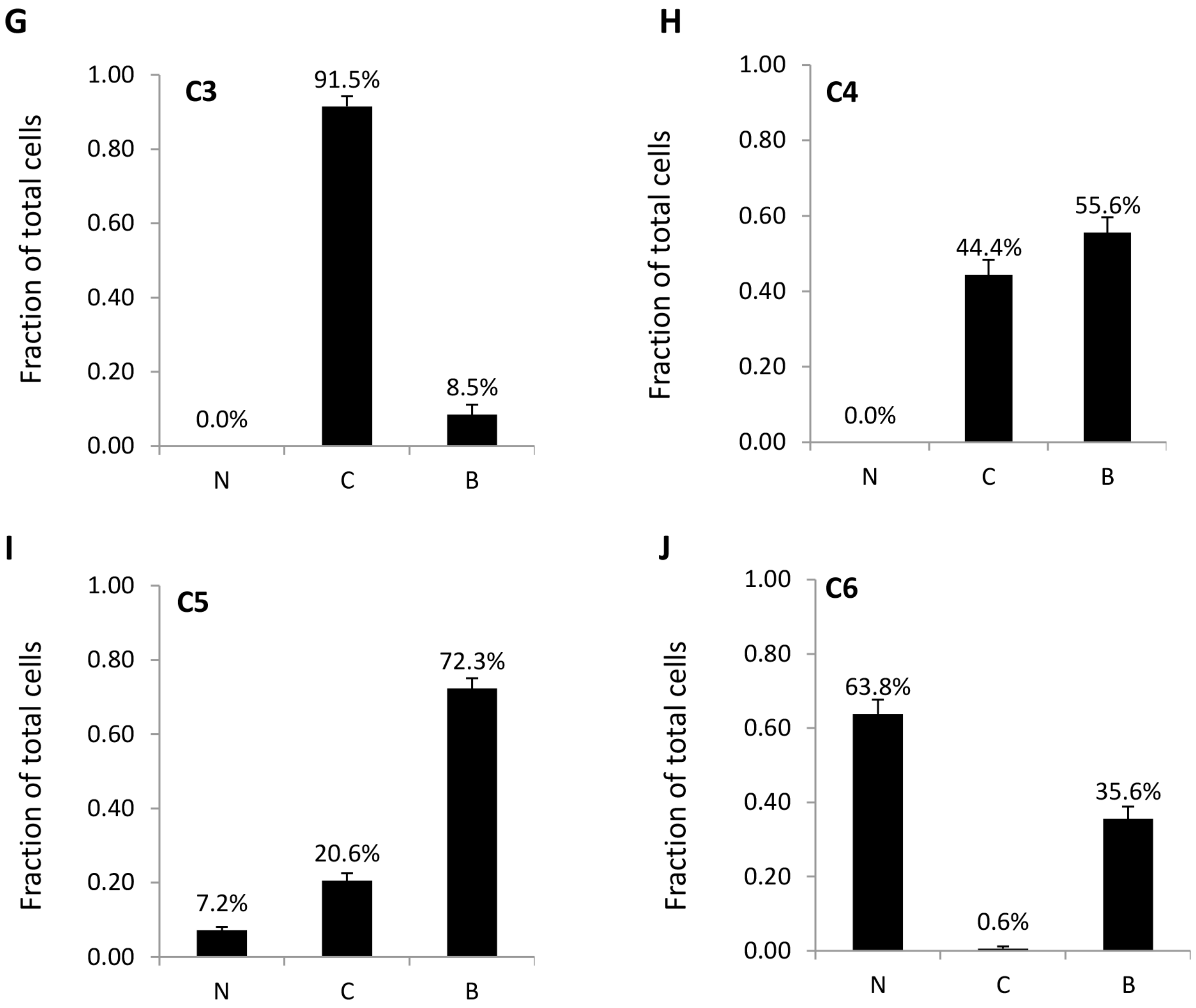

Supplementary Figure S2: Deletion analysis of TBLR1 to identify localization sequences. (**A–J**) Quantification of TBLR1 localization observed in 293T cells transfected with mutant GFP-TBLR1 constructs. Cells showing GFP expression by fluorescent imaging were counted as cytoplasmic, nuclear, or both for expression.

Supplementary Table S1: List of primers used for mutation analysis cloning

N1	F: 5′-aaggCATATGatgagtataagcagtg-3′
	R: 5′-gtacGGATCCCTActgactgatatggctttc-3′
N2	F: 5′-aaggCATATGatgagtataagcagtg-3′
	R: 5′-gtacGGATCCCTAggcatctatcagggacag-3’
N3	F: 5′-aaggCATATGatgagtataagcagtg-3′
	R: 5′-gtacGGATCCCTAagagccactGGTgctgtt-3′
N4	F: 5′-aaggCATATGatgagtataagcagtg-3′
	R: 5′-gtacGGATCCCTActgttgcttggcttcacc-3′
N5	F: 5′-aaggCATATGatgagtataagcagtg-3′
	R: 5′-gtacGGATCCCTAatcatggacacaattgtc-3′
N6	F: 5′-aaggCATATGatgagtataagcagtg-3′
	R: 5′-gtacGGATCCCTAggtatggatgcatatccc-3′
C1	F: 5′-gtacCATATGtccaatataaatggtgcc-3′
	R: 5′-aagtGGATCCCTAtttccgaaggtctaatac-3′
C2	F: 5′-gtacCATATGcctgatgtagtacaaaca-3′
	R: 5′-aagtGGATCCCTAtttccgaaggtctaatac-3′
C3	F: 5′-gtacCATATGacacagttagtacttaga-3′
	R: 5′-aagtGGATCCCTAtttccgaaggtctaatac-3′
C4	F: 5′-gtacCATATGtttccttttcattcagca-3′
	R: 5′-aagtGGATCCCTAtttccgaaggtctaatac-3′
C5	F: 5′-gtacCATATGttgcaagcacataataaa-3′
	R: 5′-aagtGGATCCCTAtttccgaaggtctaatac-3′
C6	F: 5′-gtacCATATGttgacaaaacaccaagag-3′
	R: 5′-aagtGGATCCCTAtttccgaaggtctaatac-3′
N7	F: 5′-aaggCATATGatgagtataagcagtg-3′
	R: 5′-aagtGGATCCCTAagactctattggtcgaccatca-3′
C7	F: 5′-aaggCATATGctgtccctgatagatgccgtaa-3′
	R: 5′-aagtGGATCCCTAtttccgaaggtctaatac-3′
C8	F: 5′-ttacgaCATATGgatggtcgaccaatagag-3′
	R: 5′-aagtGGATCCCTAtttccgaaggtctaatac-3′
Leu81Ala	5′-cggcatctatcagggacgcagactctattggtcgac-3′
	5′-gtcgaccaatagagtctgcgtccctgatagatgccg-3′
Leu83Ala	5′-ggcattacggcatctatcgcggacagagactctattgg-3′
	5′-ccaatagagtctctgtccgcgatagatgccgtaatgcc-3′

F: Forward; R: Reverse.

Forward and reverse primers used to create pCDNA GFP N1-N7, C1-C8, and site-directed mutation primers to modify leucine to alanine residues.
